# Smoking behavior and smoking index as prognostic indicators for patients with esophageal squamous cell carcinoma who underwent surgery: A large cohort study in Guangzhou, China

**DOI:** 10.18332/tid/117428

**Published:** 2020-02-12

**Authors:** Lili Liu, Chaoyun Huang, Wei Liao, Shuwei Chen, Shaohang Cai

**Affiliations:** 1 State Key Laboratory of Oncology in South China, Guangzhou, China; 2 Collaborative Innovation Center for Cancer Medicine, Guangzhou, China; 3 Department of Pathology, Sun Yat-sen University Cancer Center, Guangzhou, China; 4 Intensive Care Unit, Sun Yatsen University Cancer Center, Guangzhou, China; 5 Department of Head and Neck Surgery, Sun Yat-sen University Cancer Center, Guangzhou, China; 6 Department of Infectious Diseases and Hepatology Unit, Nanfang Hospital, Southern Medical University, Guangzhou, China

**Keywords:** smoking, overall survival, esophageal squamous cell carcinoma, prognostic marker

## Abstract

**INTRODUCTION:**

This study aimed to evaluate the association between smoking and smoking index with clinical outcomes of esophageal squamous cell carcinoma patients.

**METHODS:**

This is a retrospective analysis conducted on consecutive patients with esophageal carcinoma who underwent esophagectomy from January 2005 to December 2010. All patients had pathologically confirmed esophageal squamous cell carcinoma. The association between smoking and sociodemographic characteristics with overall survival and disease-free survival was analyzed. Serum carcinoembryonic antigen was measured using an electrochemiluminescence immunoassay.

**RESULTS:**

A total of 944 patients were enrolled. Kaplan–Meier analysis indicated that esophageal squamous cell carcinoma patients who smoked had a significantly worse prognosis in terms of both overall survival (p=0.007) and disease-free survival (p= 0.010). Multivariate analysis demonstrated that age (p=0.001), carcinoembryonic antigen (p=0.012), tumor-node-metastasis (TNM) staging (p<0.001) and smoking (p=0.048) were independently correlated with overall survival, while only TNM stage (p<0.001) and smoking (p=0.041) were identified as independent factors of disease-free survival. We divided the smoking population into two groups (smoking index <400 and ≥400). Kaplan–Meier survival analysis indicated that a smoking index <400 was associated with a significantly better prognosis in terms of both overall survival (p=0.003) and favorable disease-free survival (p=0.032). Multivariate analysis showed that age (p<0.001), TNM staging (p<0.001), and smoking index (p=0.025) were independent factors of overall survival, whereas for disease-free survival, only TNM stage (p=0.001) and smoking index (p=0.025) were identified.

**CONCLUSIONS:**

Overall survival was significantly associated with smoking in esophageal squamous cell carcinoma patients. For esophageal squamous cell carcinoma patients who smoke, a higher smoking index is associated with worse clinical outcomes. Therefore, smoking may be used as a predictive indicator for pretreatment evaluation and adjustment of treatment regimen.

## INTRODUCTION

Esophageal cancer (EC) is a common malignancy worldwide^1^. According to the International Agency for Research on Cancer, EC is one of the eight most common malignancies^2,3^. EC is also one of the six most deadliest tumors in the world^2,3^. China has a high risk of EC, and the mortality rate of EC ranks fourth in all malignant tumors, followed by lung cancer, stomach cancer, and liver cancer^4,5^. EC consists of two primary pathological types: squamous cell carcinoma and adenocarcinoma. Worldwide, the incidence of esophageal squamous cell carcinoma (ESCC) is slightly higher than that of adenocarcinoma. Between these two types of cancer, adenocarcinoma mainly occurs in Western countries, while in East Asian countries and China, EC is primarily squamous cell carcinoma^5^.

The pathogenesis of EC is complex and not fully understood. Epidemiological studies have confirmed that smoking is an important risk factor of ESCC^6,7^. The Center for International Cancer Research categorizes tobacco and alcohol as Class I carcinogens with sufficient evidence to prove their carcinogenicity. Several studies have shown that smoking is an important risk factor for the development of EC, especially ESCC^8-10^. Zambon et al.^11^ conducted a case-control study on the relationship between EC and smoking in three regions of northern Italy, involving 275 patients with ESCC and 593 control. They found that smoking was a strong risk factor for the development of EC. The incidence of ESCC was 7 times higher than that of non-smokers in the study population^11^.

One of the most common ways to model smoking is by dividing subjects into never and current smoker categories^12^. Compared to never smoking, current smoking was associated with increased cardiovascular disease risk^13^. However, one way that heterogeneity can enter into smoking status categories is via smoking amount, specifically cigarettes per day. Previous studies suggest that there is an increased risk of death due to coronary heart disease with increased amount^14^. This is a common way of adjusting for smoking^12^. Pack-years is a cumulative measure of smoking and is generally calculated by multiplying average packs smoked per day by the duration of smoking in years^12^. Similar to pack-years, we used the smoking index, which integrates the duration of smoking and the number of cigarettes smoked per day. Thus, the smoking index is equal to the daily tobacco intake multiplied by the duration of smoking in years.

Tobacco has been confirmed to be associated with the occurrence of many tumors^15,16^. Tobacco smoking is well known to promote the development of EC, irrespective of the pathological type^17^. A previous review noted that tobacco smoking induces a more malignant tumor phenotype by increasing the cell proliferation, cell mobility, as well as angiogenesis, and by activating cellular pro-survival pathways^18^. However, the role of smoking in the prognosis of ESCC still lacks evidence. Studies have also found that tobacco induces increased serum carcinoembryonic antigen (CEA). CEA is a promising tumor biomarker in patients with ESCC. Studies have indicated that CEA is a predictor of OS for prognosis in ESCC^19,20^.

Patients with esophageal squamous cell cancer usually have a poor prognosis. Despite the various advances in multimodal treatment strategies, the 5-year overall survival (OS) rate is still poor^1^. In addition, because of differences among patients of different ethnic and geographical origins, the association and predictive value of smoking need to be tested in different populations. Hence, the current study aims to determine the association of smoking with OS in ESCC patients.

## METHODS

### Patient selection and data collection

This is a retrospective analysis conducted on consecutive patients with EC who underwent esophagectomy at Sun Yat-sen University Cancer Center, Guangzhou, China, between January 2005 and December 2010. The study was approved by the Medical Ethics Committee and Clinical Trial Review Committee of the Cancer Center. All procedures followed were in accordance with the ethical standards of the responsible committee on human experimentation and with the Helsinki Declaration of 1975, as revised in 2008. Informed consent was obtained from all patients for inclusion in the study.

A total of 944 patients were enrolled. All patients had pathologically confirmed ESCC. Patients were excluded if: 1) they had an additional carcinoma, 2) they underwent palliative esophagectomy, or 3) their clinical data were not complete. All data were collected from medical records, and survival data were obtained from the Cancer Center’s follow-up registry. The pathologic staging of tumors for patients was based on the 8th edition of the American Joint Committee on Cancer (AJCC) tumor-node-metastasis classification^6^. In our study, all patient information was extracted from medical records. To control for bias, patients were included in the study consecutively. We included all patients with complete data in the analysis. The flow chart is shown in Supplementary file Figure 1.

**Figure 1:**
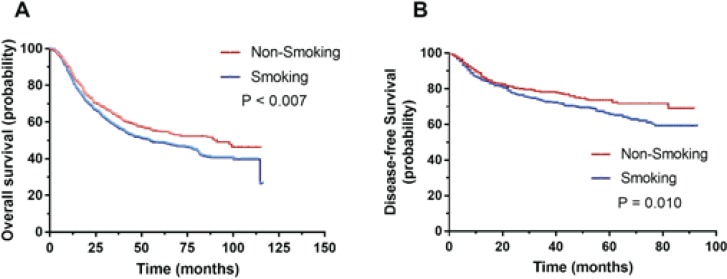
Association of smoking index and clinical outcomes in smoking patients: (A) Kaplan–Meier analysis indicated that ESCC patients with smoking were accompanied with significantly worse prognosis in terms of overall survival (p=0.007); (B) Non-smoking was positively correlated with favorable disease-free survival (p=0.010 )

### Study endpoints

In this study, the primary endpoint was disease-free survival (DFS), and the secondary endpoint was OS. DFS was defined from the date of surgery to the date of disease locoregional relapse or distant metastasis or death from any other cause. OS was defined as the interval from the date of surgery to the date of death from any cause.

### Laboratory tests

Serum carcinoembryonic antigen (CEA) was measured using a commercially available electrochemiluminescence immunoassay (Cobas E602-2, Hoffmann-La Roche Ltd, Pleasanton, CA, USA). The normal values of CEA are <5 ng/mL. Serum albumin (ALB) and globulin (GLB) levels were determined using automated techniques (LABOSPECT 008, Hitachi-hitec Globe Ltd, Tokyo, Japan). The normal ALB and GLB levels are 40–55 g/L and 20–30 g/L, respectively.

The tumor size and differentiation were reported by at least three experienced pathologists who were not informed about the patients’ preoperative conditions.

### Sociodemographic information

Sociodemographic and smoking characteristics such as gender, age, duration of smoking, daily tobacco intake, and family history, were collected from all participating subjects by a questionnaire. The smoking index integrates the duration of smoking and the number of cigarettes smoked per day, similar to pack-years.

Smoking index = daily tobacco intake × duration of smoking.

All subjects finished the questionnaire surveys in a quiet room without any interference or disruptions. Professional staff members were available to answer ques-tions if any problems occurred in understanding the survey questions^21^.

To further explore the relationship between different amounts of smoking and clinical features, we divided the smoking population into two groups, those with a smoking index <400 and those with ≥400.

### Statistical analysis

Categorical variables were calculated using Fisher’s exact test and chi-squared test, while continuous variables were analyzed using Student’s t-test. Multivariable logistical regression was performed to assess patient and tumor characteristics. All endpoints were estimated by the Kaplan–Meier method and compared using the log-rank test. Multivariable survival analyses were performed using the Cox proportional hazards model to identify important prognostic factors for OS and DFS. Two-sided p-values of <0.05 were considered statistically significant. All analyses were performed using SPSS20.0 software (SPSS Inc., Chicago, IL, USA).

## RESULTS

### Baseline sociodemographic and clinical characteristics

Consecutive patients with EC who underwent esophagectomy between January 2005 and December 2010 were enrolled (n=944). Patient outcomes were recorded from January to December 2015. The detailed baseline characteristics according to smoking behavior are listed in Table 1. Serum GLB levels in ESCC patients who smoked (26.3±4.9) were significantly lower than in ESCC patients who did not smoke (28.1±5.6) (p<0.001). The serum level of CEA in the smoking group (3.5±2.8 ng/mL) was significantly higher than in the non-smoking group (2.8±3.8 ng/mL) (p=0.002).

**Table 1:** Baseline demographic and clinical characteristics of ESCC patients, Guangzhou, 2005–2010 (N=944 )

*Characteristics*	*Smokers*	*Non-smokers*	*p*
**Sample size**	608	336	
**Gender**, M/F	599/9	127/209	<0.001
**Age** (years)	58.1±8.76	59.0±9.57	0.135
**ALB**, g/L	42.9±4.2	43.5±5.1	0.062
**GLB**, g/L	26.3±4.9	28.1±5.6	<0.001
**CEA**, ng/mL	3.5±2.8	2.8±3.8	0.002
**Tumor site**, n			0.031
Upper	64	25	
Middle	359	227	
Distal	185	84	
**Tumor size**, cm	4.6±1.9	4.3±1.8	0.003
**Differentiation**			0.529
G1	128	62	
G2	291	160	
G3	189	114	
**TNM**, n			0.008
Stage 1	59	41	
Stage 2	260	173	
Stage 3	277	119	
Stage 4	12	3	

Numbers with ± represent mean ± SD.

The proportion of ESCC patients in the TNM stage was significantly different between the two groups. In the smoking group: 59, 260, 277 and 12 patients were in stages 1, 2, 3 and 4, respectively. Whereas in the non-smoking group: 41, 173, 119 and 3 patients were in stages 1, 2, 3 and 4, respectively (p=0.008). Details are shown in Table 1.

### Association of smoking and clinical outcomes

To determine the prognostic impact of smoking on ESCC patients, Kaplan–Meier survival analysis was performed. Kaplan–Meier analysis indicated that ESCC patients who smoked had a significantly worse prognosis in terms of OS (p=0.007). The non-smoking group was positively correlated with favorable DFS (p=0.010) (Figure 1).

In the smoking group, a total of 147 patients (24.2%) had post-operative complications, compared with 77 in the non-smoking group (22.9%). Although the rate of post-operative complications was relatively higher in the smoking group, there was no significant difference between the two groups (p=0.655). The most common complications were anastomotic fistula (n=55), anastomotic stenosis (n=40), pneumonia (n=10), and other complications. At the end of enrollment of the study, the smoking group had a mortality rate of 54.7% (n=333), significantly higher than the non-smoking group with 47.0% (n=158) (p=0.022).

### Analysis of the independent factors for OS and DFS

Cox regression analysis was used to determine the associations between clinical features with OS. As shown in Tables 2 and 3, multivariate analysis showed that age (hazard ratio, HR=1.018; 95% CI: 1.008–1.028; p=0.001), CEA (HR=1.033; 95% CI: 1.007–1.060; p=0.012), TNM staging (HR=1.983; 95% CI: 1.714–2.293; p<0.001), and smoking (HR=1.214; 95% CI: 1.002–1.471; p=0.048) were independently correlated with OS (Table 2). Similarly, multivariate analysis showed that only TNM stage (HR=1.453; 95% CI: 1.208–1.747; p<0.001) and smoking (HR=1.597; 95% CI: 1.009–2.110; p=0.041) were independently correlated with DFS (Table 3).

**Table 2:** Analysis of the independent factors for overall survival of all ESCC patients, Guangzhou, 2005–2010 (N=944 )

*Variables*	*Univariate analysis*			*Multivariate analysis*		
	*HR*	*95% CI*	*p*	*HR*	*95% CI*	*p*
Gender	1.113	0.816–1.520	0.499			
Age	1.016	1.006–1.027	0.002	1.018	1.008–1.028	0.001
ALB	0.992	0.972–1.013	0.471			
GLB	1.013	0.995–1.032	0.161			
CEA	1.034	1.008–1.061	0.009	1.033	1.007–1.060	0.012
Tumor site	0.976	0.835–1.142	0.764			
Tumor size	1.027	0.977–1.080	0.299			
Differentiation	1.098	0.967–1.245	0.150			
TNM	1.933	1.663–2.247	<0.001	1.983	1.714–2.293	<0.001
Smoking index	1.323	1.009–1.735	0.043	1.214	1.002–1.471	0.048

**Table 3:** Analysis of the independent factors for disease-free survival of all ESCC patients, Guangzhou, 2005–2010 (N=944 )

*Variables*	*Univariate analysis*			*Multivariate analysis*		
	*HR*	*95% CI*	*p*	*HR*	*95% CI*	*p*
Gender	0.755	0.476–1.198	0.233			
Age	0.986	0.973–1.001	0.058			
ALB	0.990	0.960–1.020	0.493			
GLB	1.002	0.977–1.027	0.896			
CEA	1.028	0.992–1.065	0.125			
Tumor site	0.868	0.702–1.074	0.194			
Tumor size	0.969	0.903–1.039	0.377			
Differentiation	1.089	0.916–1.296	0.333			
TNM	1.438	1.188–1.740	<0.001	1.453	1.208–1.747	<0.001
Smoking index	1.347	1.024–1.964	0.002	1.597	1.209–2.110	0.001

### Factors associated with smoking index

To further explore the relationship between different amounts of smoking and clinical features, we divided the smoking population into those with a smoking index <400 and those with ≥400. For ESCC patients who smoked, the results showed that older age was observed among those with a smoking index ≥400 (p=0.028). The proportion of ESCC patients in the TNM stage was significantly different between the two groups. In the group with a smoking index <400, there were: 12, 1, 68 and 43 patients in stages 1, 2, 3 and 4, respectively. Whereas in the group with a smoking index ≥400, there were: 47, 192, 234 and 10 patients in stages 1, 2, 3 and 4, respectively (p=0.024). Details are shown in Table 4.

**Table 4:** Factors associated with smoking index among smoking ESCC patients, Guangzhou, 2005–2010 (N=944 )

*Characteristics*	*Smoking index <400*	*Smoking index ≥400*	*p*
**Sample size**	125	483	
**Gender**, M/F	120/5	479/4	0.009
**Age** (years)	56.4±10.9	58.4±8.2	0.028
**ALB**, g/L	43.0±4.4	42.8±4.1	0.776
**GLB**, g/L	26.1±4.5	26.3±5.1	0.601
**CEA**, ng/mL	3.1±2.6	3.6±2.8	0.094
**Tumor site**, n			0.705
Upper	15	49	
Middle	70	289	
Distal	40	145	
**Tumor size**, cm	4.7±1.9	4.5±1.8	0.495
**Differentiation**			0.016
G1	17	111	
G2	73	218	
G3	35	154	
**TNM**, n			0.024
Stage 1	12	47	
Stage 2	68	192	
Stage 3	43	234	
Stage 4	2	10	

Numbers with ± represent mean ± SD.

### Association of smoking index and clinical outcomes

**Figure 2:**
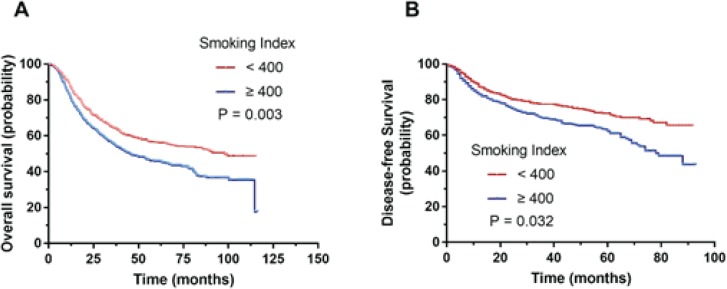
Survival analyses for revealing the prognostic value of smoking index: (A) Kaplan–Meier survival analysis was performed and results indicated that smoking index <400 were accompanied with significantly better prognosis, in terms of overall survival (p=0.003 ); (B) ESCC patients with smoking index <400 have favorable disease-free survival (p=0.032 )

We further determined the prognostic impact of smoking index on ESCC patients who smoke. Kaplan–Meier survival analysis was performed, and results indicated that a smoking index <400 was accompanied by a significantly better prognosis, in terms of OS (p=0.003) and favorable DFS (p=0.032) (Figure 2).

### Analysis of the independent factors for clinical outcomes of patients with smoking

As shown in Table 5, and in Supplementary file Table 1, multivariate analysis showed that age (HR=1.022; 95% CI: 1.009–1.035; p<0.001), TNM staging (HR=2.047; 95% CI: 1.706–2.456; p<0.001), and smoking index (HR=1.070; 95% CI: 1.008–1.135; p=0.025) were independently correlated with OS (Table 5). While for DFS, multivariate analysis showed that only TNM stage (HR=1.435; 95% CI: 1.159–1.777; p=0.001) and smoking index (HR=1.070; 95% CI: 1.008–1.135; p=0.025) were independently correlated with DFS (Supplementary file Table 1).

**Table 5:** Analysis of the independent factors for overall survival among smoking ESCC patients, Guangzhou, 2005–2010 (N=944 )

*Variables*	*Univariate analysis*			*Multivariate analysis*		
	*HR*	*95% CI*	*p*	*HR*	*95% CI*	*p*
Gender	1.825	0.839–3.969	0.129			
Age	1.016	1.003–1.030	0.017	1.022	1.009–1.035	<0.001
ALB	0.983	0.991–1.073	0.204			
GLB	1.011	0.988–1.033	0.354			
CEA	1.031	0.991–1.073	0.127			
Tumor site	0.931	0.773–1.122	0.455			
Tumor size	1.065	1.003–1.132	0.041			
Differentiation	1.075	0.920–1.257	0.363			
TNM	1.964	1.627–2.370	<0.001	2.047	1.706–2.456	<0.001
Smoking index	1.054	1.005–1.354	0.004	1.070	1.008–1.135	0.025

## DISCUSSION

The results of this study show that in patients diagnosed with ESCC in the Cancer Centre, OS was significantly associated with smoking as well as with recognized risk factors including age and TNM stage. In addition, we found that ESCC patients with a higher smoking index had worse clinical outcomes. For all ESCC patients, age, CEA, TNM staging and smoking were independently correlated with OS. While for ESCC patients who smoked, only age, TNM staging and smoking index were identified as independent risk factors correlated with OS. The results show that smoking is not only a risk factor for developing ESCC but also an independent risk factor for poor prognosis. Moreover, we found that the higher the smoking amount, the worse the prognosis.

ESCC is the major type of EC in China^22^. The histological type of ESCC accounted for more than 80% of EC patients^22,23^. To avoid further bias, the study only included ESCC patients. As EC is one of the most common malignancies, its incidence is generally higher in male than female patients^23^, and the ratio of males to females is about 2:1. It is generally believed that EC is a malignant tumor formed by the interaction of multiple factors and multiple genes^1,8,10^. Although many studies have conducted in-depth research on the prevention and treatment of EC and achieved results, the prognosis of EC is poor because of its concealed and invasive nature. Identifying the risk factors for EC and then using risk stratification and different interventions can effectively reduce the mortality associated with EC.

It is well established that smoking is an important factor in the development of EC^10,11^. The unique chemical compounds of tobacco can regulate the expression of a large number of genes, including genes encoding tumor invasion and metastasis, which can lead to changes in tumor molecular physiology^15,16,22^. However, there is only little evidence of the association between smoking and the prognosis of EC, and the conclusions are inconsistent. Situ et al.^24^ found that smoking before ESCC diagnosis is an independent factor of prognosis. Our study confirmed that smoking is an independent prognostic factor for ESCC. In addition, we confirmed that smoking has an amount-dependent effect on the prognosis of ESCC. The higher the smoking index, the worse the patient’s prognosis. However, it is worth noting that the harm of secondhand smoke has been confirmed by many studies^25-29^. In ESCC, there is no research on the relationship between secondhand smoke and EC. How secondhand smoke affects the occurrence and development of EC requires further research and confirmation. The relationship between tobacco and occurrence of ESCC has been confirmed in many studies. However, for the prognosis of patients with esophageal cancer, especially in ESCC, which is a cancer with low incidence in the West, the prognostic impact of tobacco in ESCC still lacks medical evidence. In this study, we demonstrated that tobacco is an independent risk factor for poor prognosis in ESCC patients. The higher smoking index, the worse the prognosis of ESCC patients.

In relation to surgical group, many factors could interfere in the outcome: surgical access (thoracoscopic is related to more respiratory complications than transhiatal approach), number of retrieved lymph nodes (as a marker of sufficient lymphadenectomy), and complications^30^. However, in this study, multivariate analysis suggested that smoking and smoking index were independent risk factors for poor prognosis in ESCC patients. This means that regardless of the surgical access or complications, the smoking and smoking index are still independently affecting the prognosis of ESCC.

In our study, we found that smokers’ CEA levels were significantly higher than those of non-smokers. CEA is a classic tumor marker. Studies also found smoking can raise CEA levels^31,32^. Fukuda et al.^33^ showed that CEA-positive patients were more often heavy smokers. This result is consistent with our results. However, the molecular mechanism by which tobacco increases CEA levels remains unclear. In our study, we also found that smokers have lower globulin levels. This is similar to the results of a previous study, which reported that smoking induces changes such as decreased leukocyte chemotaxis, decreased production of globulins, and impaired phagocytosis^34^. Furthermore, our study found that smokers and non-smokers have significant differences in the tumor sites. The proportion of esophageal cancer in distal esophagus among smokers was significantly higher than in non-smokers. Moreover, the tumor size of esophageal cancer of smokers is significantly larger than that of non-smokers, as similarly reported^35^. Whether tobacco promotes the proliferation of esophageal cancer cells is an interesting but still unclear question.

Neoadjuvant chemoradiotherapy has been proposed for ESCC patients^36^. A recent study has revealed that neoadjuvant chemoradiotherapy followed by esophagectomy has a significant survival benefit compared to esophagectomy alone^37^. However, following neoadjuvant chemoradiotherapy, a certain proportion of ESCC patients do not respond to chemoradiotherapy^38,39^. Studies have found that neoadjuvant therapy is not associated with better results than surgery alone in ESCC patients^40,41^. However, the patients included in this study did not receive neoadjuvant chemoradiotherapy. But whether smoking has value to predict the benefit after neoadjuvant chemoradiotherapy is a question waiting to be answered.

### Strengths and limitations

The current study has some limitations. First, some other baseline characteristics, variations in therapy regimens, as well as interactions between variables may have caused bias in the results. This study confirms that the smoking and smoking index predicts poor prognosis in ESCC patients after surgery, but further studies are still needed to confirm the relationship between smoking and prognosis in ESCC patients who have not received surgery. Despite this, the results showed a strong possibility for the use of smoking as a predictive marker for ESCC patient prognosis after surgery.

## CONCLUSIONS

The results of the present study showed that smoking is independently associated with OS in ESCC patients. Therefore, smoking may be used as a predictive indicator for pretreatment evaluation and adjustment of treatment regimen.
