# Supplementary figures and images for: Smoking behavior and smoking index as prognostic indicators for patients with esophageal squamous cell carcinoma who underwent surgery: A large cohort study in Guangzhou, China
Source: Tob Induc Dis. 2020 Feb 12;18:9. doi: 10.18332/tid/117428 (PMC7019194; doi:10.18332/tid/117428)

**Supplementary file, Figure 1**

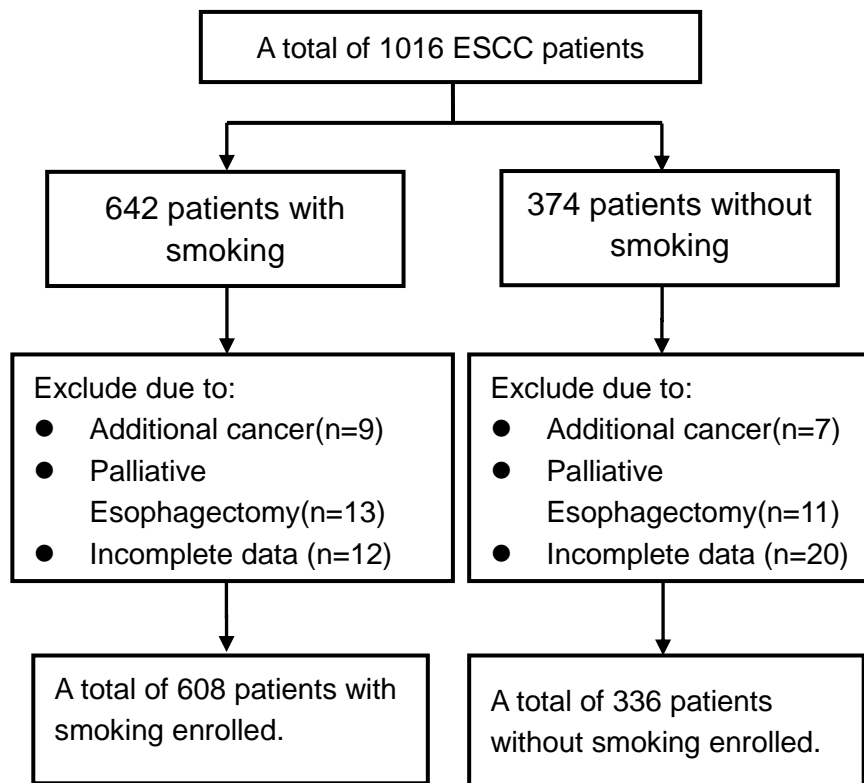

Supplement: Supplementary file 1 [file TID-18-09-s1.pdf]
