# Supplementary material for: Smoking behavior and smoking index as prognostic indicators for patients with esophageal squamous cell carcinoma who underwent surgery: A large cohort study in Guangzhou, China
Source: Tob Induc Dis. 2020 Feb 12;18:9. doi: 10.18332/tid/117428 (PMC7019194; doi:10.18332/tid/117428)
Supplement: Supplementary file 2 [file TID-18-09-s2.pdf]

Supplementary Table 1. Analysis of the independent factors for disease-free survival among smoking ESCC patients, Guangzhou, 2005-2010 (N=944).

Variables	Univariate analysis			Multivariate analysis		
	HR	95%CI	P	HR	95%CI	P
Gender	0.504	0.069-3.652	0.497			
Age	0.985	0.968-1.003	0.099			
ALB	0.980	0.945-1.016	0.276			
GLB	1.006	0.977-1.037	0.688			
CEA	1.016	0.966-1.070	0.531			
Tumor site	0.809	0.634-1.032	0.089			
Tumor size	0.998	0.920-1.083	0.963			
Differentiation	1.084	0.884-1.328	0.438			
TNM	1.365	1.089-1.712	0.007	1.435	1.159-1.777	0.001
Smoking index	1.455	1.085-2.150	0.032	1.070	1.008-1.135	0.025

© 2020 Liu L. et al.
